# Electromyographic Analysis of Masticatory and Accessory Muscles in Subjects With Implant-Supported Fixed Prostheses: A Three-Arm Comparative Clinical Study

**DOI:** 10.7759/cureus.33969

**Published:** 2023-01-19

**Authors:** Vaibhava Raaj, Sakshi Raina, Romshi Raina, Abhishek ., Madhuri Kumari, Anusha .

**Affiliations:** 1 Department of Periodontics, Exservicemen Contributory Health Scheme Polyclinic, Ara, IND; 2 Department of Orthodontics, Exservicemen Contributory Health Scheme Polyclinic, Samastipur, IND; 3 Department of Public Health Dentistry, National Resource Centre for Oral Health and Tobacco Cessation, Maulana Azad Institute of Dental Sciences, New Delhi, IND; 4 Department of Oral and Maxillofacial Surgery, Oro Care Facial Trauma Centre, Patna, IND; 5 Department of Public Health Dentistry, Sadar Hospital, Arwal, IND; 6 Department of Oral Medicine and Radiology, Oro Care Facial Trauma Centre, Patna, IND

**Keywords:** maximum voluntary contraction, implant supported prosthesis, masticatory muscles, implants, electromyography

## Abstract

Aim

This study compares the electromyographic (EMG) activity of the masticatory and accessory muscles in patients with natural teeth and those wearing full-mouth fixed prostheses supported by implants.

Method

In this study, 30 subjects of 30-69 years performed static and dynamic EMG measurements of masticatory and accessory muscles (masseter, anterior temporalis, SCM, and anterior digastric) and were divided into three groups: Group 1 (G1, Control, Dentate), comprising 10 subjects with 14 or more natural teeth (30-51 years of age); Group 2 (G2, single arch implant-supported fixed prosthesis) composed of 10 patients with unilateral edentulism who were successfully rehabilitated with implant-supported fixed prostheses in the maxilla or mandible, restoring occlusion to 12-14 teeth per arch; (39-61 years of age); and Group 3 (G3, full mouth implant-supported fixed prosthesis) with 10 subjects with completely edentulous arches with full mouth implant-supported fixed prosthesis with 12 occluding pairs of teeth (46-69 years of age). The left and right masseter, anterior temporalis, superior sagittal, and anterior digastric muscles were examined at rest, as well as maximum voluntary clenching (MVC), swallowing, and unilateral chewing. On muscle bellies, disposable, pre-gelled silver/silver chloride bipolar surface electrodes were parallel to muscle fibers. BIO-PAKeight® channels recorded electrical muscle activity (Bio-EMG III, BioResearch Associates, Inc. Brown Deer, WI).

Results

Full mouth embed upheld fixed prostheses patients had higher resting EMG activity than dentate and single curve implants. Full mouth embeds supported fixed prostheses and dentate patients had significantly different temporalis and digastric muscle mean EMG activity. Dentate people used their temporalis and masseter muscles more during the MVC than those with single-curve embedded upheld fixed prostheses limiting natural teeth or full-mouth implants. No event had the crucial item. Neck muscle differences were insignificant. All groups had higher SCM and digastric EMG activity during MVC than at rest. The single curve embed upheld fixed prosthesis group's temporalis and masseter muscles were significantly more active during gulping than the dentate and entire mouth groups. Single curve and entire mouth gulping SCM muscle EMG activity were similar. Digastric muscular EMG activity differed significantly between those with full-arch or partial-arch fixed prostheses and dentures. When instructed to bite one side, the masseter and temporalis front muscle mean EMG activity increased on the unrestricted side. Unilateral biting and temporalis muscle activation were comparable between groups. For the masseter muscle, the mean EMG was also higher on the functioning side, with no truly large differences between the three groups except for right-side biting when comparing the dentate and full mouth embed upheld fixed prosthesis groups and the single curve and full mouth groups.

Conclusion

The temporalis muscle activity difference was statistically significant in the full mouth implant-supported fixed prosthesis group. The three groups' static (clenching) sEMG analysis showed non-significant temporalis and masseter muscle activity increases. Full mouth swallowing increased digastric muscle activity. All three groups had similar unilateral chewing muscle activity except for the working side masseter muscle.

## Introduction

Tight coordination with breathing, mastication, and swallowing make up the bulk of oral motor activity [1]. Normal human contact relies heavily on the oral and facial structures because of the psycho-physiological functions of speech and facial expression. Some of the exact regulatory mechanisms necessary for such oral motor activities are poorly known, and oral conditions and clinical dental treatments may have direct and indirect effects on them [1]. Mastication is difficult because it involves a network of involuntary and automatic motor pathways, pattern generators in the brain, and receptors that all interact with one another (exterior-, proprio-, and viscera-receptors). Replacing teeth in humans may be done using a fixed prosthesis supported by the patient's remaining teeth, the alveolar mucosa, or osseointegrated implants. A permanently implanted prosthesis is another viable alternative. In conclusion, implant-supported prostheses have the potential to restore oral function and perform better than conventional full dentures in terms of chewing performance assessed using both subjective and objective metrics [2].

Implants' ability to preserve the normal process of remodeling and prevent unwanted bone loss are two of their most important advantages, both of which contribute significantly to the preservation of the stomatognathic system [3]. That kind of therapy has the potential to affect the standard of living throughout the world. Patients with missing teeth who get implant-supported prostheses, for example, are able to eat a healthier diet. These foods, which are abundant in fiber and important vitamins, must be a part of any healthy diet [4]. The neuromuscular system may be intimidating to investigate from a clinical perspective because of the sheer number of muscle groups present, the majority of whose performance is typically difficult to assess with any degree of precision. However, there are obvious indicators of proper operation. Initially, it is best to unwind the muscles used for chewing whenever possible. And second, both sides should find it just as simple to engage in robust, pleasant mastication (alternately) [5]. After establishing centric relation, the clinician should be able to open and close the jaw freely to and from the vertical dimension without experiencing hypertonic masticatory antagonists [5]. Surface electromyography (sEMG) is a common method for assessing the responsiveness of the musculature of the stomatognathic system to both stationary and moving stimuli. sEMG techniques and markers have been developed to evaluate the harmony of masticatory muscles in patients and in controlled healthy participants during maximal voluntary teeth clenching and chewing [6]. EMG has been used in dentistry for a while now, and in that time, devices, electrodes, and processes have all been refined and standardized to the point that recorded data can be statistically assessed. This diagnostic tool integrates traditional qualitative data with new quantitative measurements [7].

EMG activity of masticatory muscles has been extensively studied in both in vivo (using implanted electrodes) and ex vivo (using surface recordings) studies. Using modern surface EMG equipment, scientists can evaluate the performance of several muscles involved in swallowing, chewing, and head orientation (typically the masseter, temporalis anterior and posterior, digastric anterior, and sternocleidomastoid) [8]. If proper procedures are followed, recorded measurements are reliable in terms of both precision and consistency [7]. Symmetry assessments and analyses of activation patterns in the various masticatory muscles have been combined with EMG potentials. The eight-channel BIO-PAK® device was used to capture the subjects' EMG activity (Bio-EMG III, BioResearch Associates, Inc. Brown Deer, WI). Data is displayed visually and saved to magnetic media through a direct connection between the instrument and a personal computer. The device has been previously validated, showing high consistency of surface EMG readings using a technique quite similar to that used here [9]. Although this situation is common in clinical practice, previous studies did not evaluate the muscle activity of individuals with single arch implant-supported fixed prostheses versus natural dentition or entire mouth implant-supported fixed prostheses versus natural dentition. Also, few studies have looked at how masticatory and auxiliary (sternocleidomastoid and digastric) muscles respond to fixed implant-assisted prosthetic reconstructions in both static and dynamic activities.

## Materials and methods

Patient recruitment

Thirty subjects who were screened clinically for inclusion and exclusion criteria, aged between 30-69 years were examined for EMG activity of masticatory and accessory muscles during static and dynamic tasks. Simple random sampling methods were used in selecting patients. Group 1 (G1, control, i.e., dentate) with 10 subjects (age: 30-51 years) with a full complement of natural dentition and at least 14 pairs of teeth; Group 2 (G2, single arch implant-supported fixed prosthesis) where the subjects included 10 people who were entirely toothless in one arch but had been rehabilitated with an implant-supported fixed prosthesis in either the maxilla or mandible; these prostheses featured 12-14 occluding pairs of teeth to mimic the patient's natural dentition (age: 39-61 years); and Group 3 (full mouth implant-supported fixed prosthesis) with a full mouth implant-supported fixed prosthesis with 12 occluding pairs of teeth was used to rehabilitate 10 subjects who were completely edentulous in both arches (age: 46-69 years).

There were four to six osseointegrated implants placed in the edentulous mandible and/or maxilla of each patient who received a fixed implant-supported prosthesis. In order to conduct an EMG test, all patients had to have been using their prostheses for at least six months. Intercuspal-centric contacts were equally distributed across all occlusal systems. Inclusion criteria were patients who demonstrated sufficient masticatory efficiency and reported being happy with their prosthesis. For both patients and control subjects, the exclusion criteria were dental and periodontal problems, muscular pain in the head, neck, or shoulders, temporomandibular joint alterations, or neck disturbances.

All participants signed a preemptive informed consent form after being fully briefed on the experimental methods to which they would be subjected. Ethical clearance was also taken from the institute with ref no: IEC/2020/1222. In addition to being risk-free and painless for the participants, the procedures also gave them the option to end the test at any time they felt it was necessary. Disposable surface electrodes, gauge pieces with ethanol, Sugarless chewing gum and computerized electromyographic machine, and BIO-PAK® System were the materials used.

Materials and Methods

During one of their routine checkups at ECHS polyclinic, Ara, an EMG was conducted to assess their muscle function. Maximum voluntary clenching (MVC), one-sided gum chewing, and swallowing were all studied using sEMG of the masseter, temporalis, SCM, and anterior digastric muscles on the right and left sides of the head and neck. All EMG data were collected by a single operator who was unaware of the patients' assignments; each participant was given a specific number that would be used in later statistical calculations of electromyographic signal analysis. The sample size was calculated using G*power 3.1.7 software (Universitat Dusseldorf, Dusseldorf, Germany).

Instrumentation

**Figure 1:**
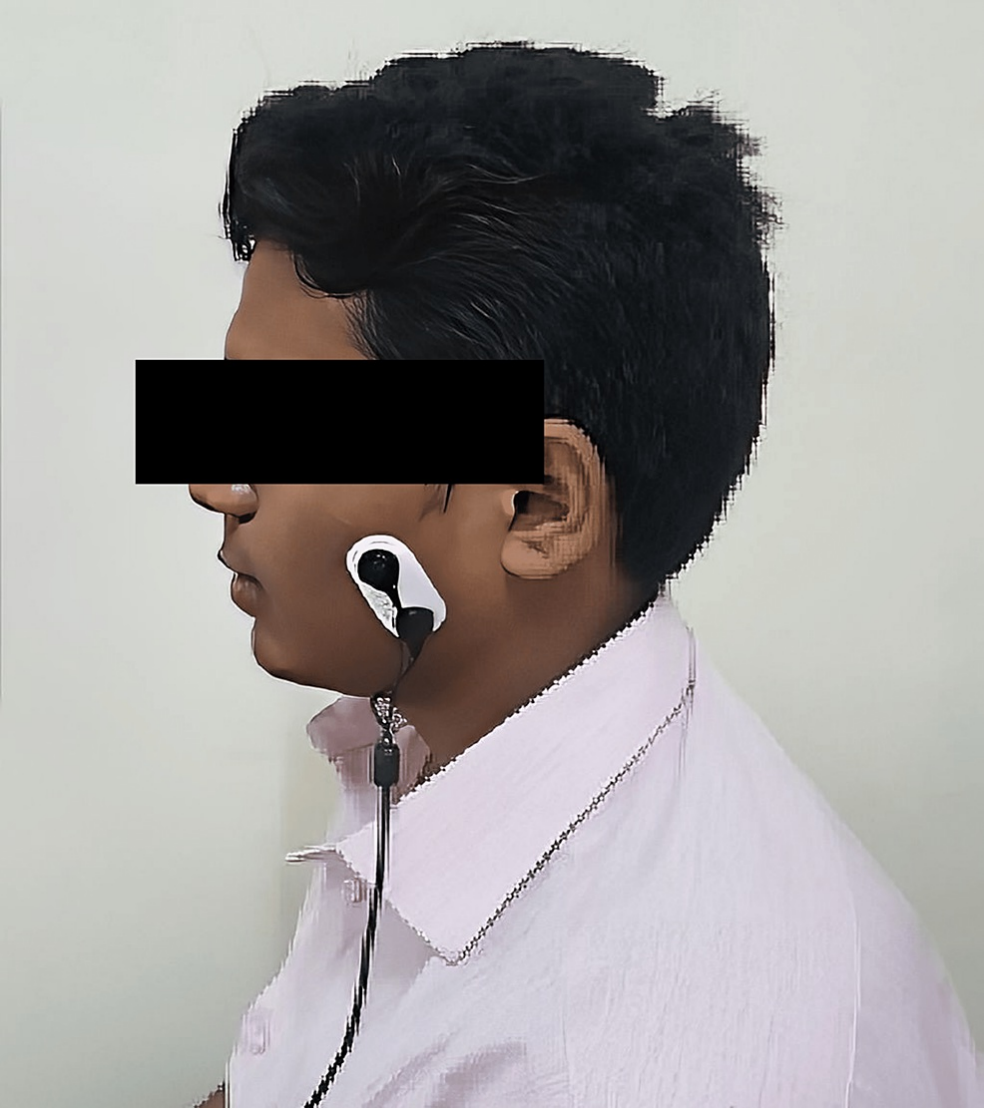
Testing the masseter muscle

The left and right masseters, anterior temporalis, splenius capitis major, and anterior digastric muscles were tested (Figure 1).

The muscle bellies were placed with disposable, pre-gelled silver/silver chloride bipolar surface electrodes parallel to the muscle fibers. The patient's name was grounded with a static-dissipating electrode. To lower the impedance of the skin for the electrodes to work, ethanol was used to clean the skin beforehand. Eight channels of a cutting-edge device called BIO-PAK® were used to capture electrical muscle activity. A differential speaker with a high familiar mode dismissal proportion, CMRR >130 dB at 60 Hz (>120 dB, 100 to 600 Hz), input impedance > 100 megohms (108), highest sign to-clamor proportion a million to 1, and wide frequency response were used to amplify the original, low-level EMG signal (gain 5000, top to top info range from 0 to 2000 V). The range of the DC voltage it can handle is -6.5 to +6.5 volts, the transfer speed is 30 to 1000 hertz (at a test rate of 2,000 hertz), the sensitivity is (0.3) microvolt (p-pp), the resolution of the A/D converter is 0.5 microvolt, the optical isolation is to the nearest millisecond, and the estimation of the quiet period is to the nearest microsecond. UL-recognized opto-couplers (UL record #E58730, E54915, or same.) The patient was isolated from the computer's AC power supply, with a maximum peak voltage (applied) of 3500 volts, a leakage current of 1 microampere, a capacitance (to the AC power line) of 2 picofarads, and a reduction in program noise of 40 dB (nominal). (On a logarithmic scale, a reduction of 40 dB in commotion sufficiency is equivalent to a reduction of almost 100%.) BioPAK was used to find a median value of over 25 milliseconds for the indicators, with muscular activity measured as the root mean square (rms) of the sufficiency (V). In order to further investigate the EMG signals, recordings were made.

Measurement protocol

EMG activity was recorded during the following: (1) The no-bite, physiological rest posture (rest), wherein after the participant reached a steady state, they rested for at least 2 minutes with their eyes closed and jaw relaxed; (2) maximum voluntary clench (clench); (3) the patient was instructed to hold the posture while swallowing, using just their teeth and very little muscle as per Ferrario et al. [7]; (4) the subject was then asked to chew sugarless chewing gum and soften it for 30 seconds, and then unilateral chewing (right side and left side chewing) was performed.

MVC data collection and analysis

The individual was then instructed to clench as tightly as possible and keep the contraction going for five seconds while the EMG activity was recorded. This was an MVC in an intercuspal position. There were three separate tests conducted. The test participants were urged by the researchers to provide their best efforts and do their very best seconds while the EMG activity was recorded. Subjects were instructed to sit with their heads unsupported during all evaluations. Each test was separated by at least three minutes, and there was also a three-minute rest interval between the recording for standardization and the tests. The program automatically chose the three-second window with the most stable r.m.s. EMG signal across all trials. The program used the numerical codes to do all computations automatically, and group assignments occurred only at the conclusion of the process. In order to provide a fair comparison between the three groups, we averaged the results from all three MVC tests that each participant took.

Gum-chewing data collection and analysis

The muscle contractions of the masseter, temporalis, splenius capitis major, and digastric were measured electromyographically when participants chewed sugarless gum on just one side of their mouths (left or right) as per Ferrario et al. [5]. All patients had their first 15 seconds of unilateral biting evaluated for EMG potential. Each muscle tested (right and left) for biting was recorded for EMG analysis, and the outcomes of both tests were compared.

Descriptive statistics

The study data were analyzed using SPSS Statistics v.22 for Windows (IBM, Corp., Armonk, NY). Within each group, descriptive statistics (mean and standard deviation) were calculated for each muscle (temporalis, masseter, sternocleidomastoid, and digastric) on the right and left side during the relaxed state, maximum voluntary clenching (MCV), swallowing, unilateral chewing (right and left side). Comparing the mean EMG data over the entire research was done using inferential statistics. To compare muscle performance between groups and across tasks, we used one-way ANOVA and Tukey's post hoc test. The level of significance (p-value) was set at 0.05.

## Results

Temporalis muscle

The mean EMG activity (μV) of the temporalis (right and left side) muscle during a relaxed state, maximum voluntary clenching, swallowing, and unilateral chewing (right and left side chewing) was recorded. Statistically, a significant difference was present in (1) The right temporalis during the relaxed state between the G2 vs G3 groups; (2) the right temporalis during swallowing between the G1 vs G2 and G2 vs G3 groups; (3) the left temporalis during the relaxed state between the G1 vs G3 and G2 vs G3 groups; (4) the left temporalis during swallowing between the G1 vs G2 and G2 vs G3 groups. However, there was no statistically significant difference between the three groups during MVC and unilateral chewing. Comparison of mean EMG values (μV) of temporalis muscle between different groups at various states using a one-way ANOVA test followed by Tukey's post hoc analysis can be found in Table 1.

**Table 1:** Comparative analysis of temporalis muscle in different groups * - statistically significant C: control group; SA: single arch group; FM: full mouth group; Lt: left; Rt: right

State	Group	N	Mean	SD	P-Value	Pairwise comparison	P-Value
Relax (R Muscle)	G1 (C)	10	4.55	2.05	0.04*	C Vs SA	0.35
	G2 (S A)	10	2.80	1.45		C Vs FM	0.55
	G3 (F M)	10	5.55	2.42		SA Vs FM	0.04*
Clench (R Muscle)	G1 (C)	10	40.85	26.30	0.20	C Vs SA	0.25
	G2 (S A)	10	24.02	3.45		C Vs FM	0.40
	G3 (F M)	10	26.11	21.45		SA Vs FM	0.95
Swallow (R Muscle)	G1 (C)	10	4.45	2.60	<0.001*	C Vs SA	<0.001*
	G2 (S A)	10	16.45	5.55		C Vs FM	0.99
	G3 (F M)	10	4.85	2.04		SA Vs FM	<0.001*
Rt. Side Chew (R Muscle)	G1 (C)	10	26.04	9.30	0.70	C Vs SA	0.90
	G2 (S A)	10	29.80	5.08		C Vs FM	0.98
	G3 (F M)	10	24.98	20.45		SA Vs FM	0.68
Lt. side Chew (R Muscle)	G1 (C)	10	18.18	6.45	0.75	C Vs SA	0.80
	G2 (S A)	10	15.06	6.70		C Vs FM	0.98
	G3 (F M)	10	16.94	14.01		SA Vs FM	0.95
Relax (L Muscle)	G1 (C)	10	4.55	2.05	0.001*	C Vs SA	0.30
	G2 (S A)	10	3.65	0.95		C Vs FM	0.04*
	G3 (F M)	10	6.85	1.90		SA Vs FM	0.001*
Clench (L Muscle)	G1 (C)	10	38.08	15.05	0.20	C Vs SA	0.20
	G2 (S A)	10	27.05	4.90		C Vs FM	0.60
	G3 (F M)	10	33.55	21.95		SA Vs FM	0.65
Swallow (L Muscle)	G1 (C)	10	5.05	2.92	0.001*	C Vs SA	<0.001*
	G2 (S A)	10	15.92	2.90		C Vs FM	0.35
	G3 (F M)	10	7.50	5.45		SA Vs FM	<0.001*
Rt. side Chew (L Muscle)	G1 (C)	10	21.45	11.10	0.29	C Vs SA	0.28
	G2 (S A)	10	15.80	5.80		C Vs FM	0.40
	G3 (F M)	10	15.95	9.82		SA Vs FM	0.99
Lt. side Chew (L Muscle)	G1 (C)	10	28.11	9.70	0.20	C Vs SA	0.20
	G2 (S A)	10	20.03	5.01		C Vs FM	0.38
	G3 (F M)	10	21.55	8.75		SA Vs FM	0.92

Masseter muscle

The mean EMG activity (μV) of the masseter (right and left side) muscle during a relaxed state, maximum voluntary clenching, swallowing, and unilateral chewing (right and left side chewing) was recorded. Statistically, a significant difference was present in (1) the right masseter during swallowing between the G1 vs G2 and G2 vs G3 groups; (2) the right masseter during right-side chewing between the G1 vs G3 and G2 vs G3 groups; (3) the left masseter during swallowing between the G1 vs G2 and G1 vs G3 groups. Right and left masseter muscular activity, and also left masseter activities during right side chewing, showed no statistically significant difference among the three groups during the relaxed state, MVC, and left side chewing. A comparison of mean EMG values (μV) of masseter muscle between different groups at various states using a one-way ANOVA test followed by Tukey's post hoc analysis can be found in Table 2.

**Table 2:** Comparative analysis of masseter muscle in different groups * - statistically significant C: control group; SA: single arch group; FM: full mouth group; Lt: left; Rt: right

State	Group	N	Mean	SD	P-Value	Pairwise comparison	P-Value
Relax (R Muscle)	G1 (C)	10	4.47	3.15	0.09	C Vs SA	0.45
	G2 (SA)	10	2.75	1.26		C Vs FM	0.60
	G3 (FM)	10	5.94	4.50		SA Vs FM	0.08
Clench (R Muscle)	G1 (C)	10	27.50	10.52	0.38	C Vs SA	0.34
	G2 (SA)	10	21.02	4.40		C Vs FM	0.59
	G3 (FM)	10	23.45	8.90		SA Vs FM	0.95
Swallow (R muscle)	G1 (C)	10	5.38	3.12	0.001*	C Vs SA	0.005*
	G2 (SA)	10	14.02	8.15		C Vs FM	0.79
	G3 (FM)	10	3.70	1.55		SA Vs FM	0.001*
Rt. Side Chew (R Muscle)	G1 (C)	10	23.01	5.98	<0.001*	C Vs SA	0.32
	G2 (SA)	10	26.60	3.44		C Vs FM	0.02*
	G3 (FM)	10	15.11	7.60		SA Vs FM	<0.001*
Lt. side Chew (R Muscle)	G1 (C)	10	16.01	3.45	0.71	C Vs SA	0.96
	G2 (SA)	10	16.65	8.80		C Vs FM	0.70
	G3 (FM)	10	18.50	6.45		SA Vs FM	0.84
Relax (L Muscle)	G1 (C)	10	3.83	2.72	0.42	C Vs SA	0.52
	G2 (SA)	10	2.97	1.15		C Vs FM	0.99
	G3 (FM)	10	4.25	2.45		SA Vs FM	0.45
Clench (L Muscle)	G1 (C)	10	31.35	18.15	0.15	C Vs SA	1.00
	G2 (SA)	10	40.04	5.60		C Vs FM	0.18
	G3 (FM)	10	20.50	13.60		SA Vs FM	0.20
Swallow (L Muscle)	G1 (C)	10	6.25	3.35	<0.001*	C Vs SA	<0.001*
	G2 (SA)	10	18.21	8.72		C Vs FM	<0.001*
	G3 (FM)	10	4.95	3.21		SA Vs FM	0.89
Rt. side Chew (L Muscle)	G1 (C)	10	23.43	3.64	0.88	C Vs SA	0.88
	G2 (SA)	10	26.96	12.81		C Vs FM	0.95
	G3 (FM)	10	25.31	26.15		SA Vs FM	0.99
Lt. side Chew (L Muscle)	G1 (C)	10	27.09	10.65	0.45	C Vs SA	1.00
	G2 (SA)	10	26.88	6.01		C Vs FM	0.53
	G3 (FM)	10	20.40	17.95		SA Vs FM	0.49

Sternocleidomastoid muscle

The average electromyographic voltage (V) of the sternocleidomastoid muscle during rest, during maximal voluntary clenching, swallowing, and unilateral chewing (right and left side chewing) was recorded. Relaxed state, maximal voluntary contractions, swallowing, and unilateral chewing showed no statistically significant differences between the three groups. 

During the movement of the mandible, the SCM as well as other masticatory muscles are both involved. During rest, maximal voluntary clenching, swallowing, and unilateral chewing, the average electromyographic (EMG) activity (V) of the sternocleidomastoid (side) muscle was measured (right and left side chewing). With respect to the relaxed state, maximal voluntary contraction, swallowing, and unilateral chewing, there was no statistically significant difference between the three groups. A comparison of mean EMG values (μV) of SC mastoid muscle between different groups at various states using a one-way ANOVA test followed by Tukey's post hoc analysis can be found in Table 3.

**Table 3:** Comparative analysis of sternocleidomastoid muscle in different groups C: control group; SA: single arch group; FM: full mouth group; Lt: left; Rt: right

State	Group	N	Mean	SD	P-Value	Pairwise comparison	P-Value
Relax (R Muscle)	G1 (C)	10	2.60	1.45	0.85	C Vs SA	1.00
	G2 (SA)	10	2.54	1.25		C Vs FM	0.91
	G3 (FM)	10	2.92	1.95		SA Vs FM	0.88
Clench (R Muscle)	G1 (C)	10	2.65	1.46	0.38	C Vs SA	0.44
	G2 (SA)	10	3.74	1.48		C Vs FM	0.47
	G3 (FM)	10	3.65	2.49		SA Vs FM	1.00
Swallow (R muscle)	G1 (C)	10	2.27	0.95	0.08	C Vs SA	0.09
	G2 (SA)	10	3.25	0.85		C Vs FM	0.98
	G3 (FM)	10	2.35	0.84		SA Vs FM	0.15
Rt. Side Chew (R Muscle)	G1 (C)	10	3.01	2.70	0.06	C Vs SA	0.13
	G2 (SA)	10	4.95	2.14		C Vs FM	0.98
	G3 (FM)	10	2.71	1.60		SA Vs FM	0.07
Lt. side Chew (R Muscle)	G1 (C)	10	3.25	2.10	0.81	C Vs SA	0.97
	G2 (SA)	10	3.50	1.49		C Vs FM	0.92
	G3 (FM)	10	2.93	1.62		SA Vs FM	0.79
Relax (L Muscle)	G1 (C)	10	2.69	1.37	0.54	C Vs SA	0.85
	G2 (SA)	10	2.91	0.92		C Vs FM	0.48
	G3 (FM)	10	3.43	1.80		SA Vs FM	0.78
Clench (L Muscle)	G1 (C)	10	2.85	2.14	0.29	C Vs SA	0.67
	G2 (SA)	10	3.71	1.19		C Vs FM	0.27
	G3 (FM)	10	4.50	3.45		SA Vs FM	0.73
Swallow (L Muscle)	G1 (C)	10	2.61	1.62	0.39	C Vs SA	0.85
	G2 (SA)	10	3.80	1.91		C Vs FM	0.40
	G3 (FM)	10	5.40	7.45		SA Vs FM	0.75
Rt. Side Chew (L Muscle)	G1 (C)	10	4.15	6.75	0.95	C Vs SA	0.99
	G2 (SA)	10	4.69	2.11		C Vs FM	1.00
	G3 (FM)	10	4.25	2.11		SA Vs FM	0.97
Lt. side Chew (L Muscle)	G1 (C)	10	4.35	4.40	0.56	C Vs SA	0.85
	G2 (SA)	10	3.55	0.67		C Vs FM	0.57
	G3 (FM)	10	2.96	2.40		SA Vs FM	0.89

Anterior belly of the digastric muscle

The mean EMG activity (μV) of the digastric (right and left side) muscle during a relaxed state, maximum voluntary clenching, swallowing and unilateral chewing (right and left side chewing). Statistically, a significant difference was present in (1) the right digastric during swallowing between the G2 vs G3 groups; (2) the left digastric during a relaxed state between the G1 vs G2 groups; (3) the left digastric during right side chewing between the G1 vs G3 groups. A comparison of mean EMG values (μV) of the digastric muscle between different groups at various states using a one-way ANOVA test was conducted followed by Tukey's post hoc analysis can be found in Table 4.

**Table 4:** Comparative analysis of digastric muscle in different groups * statistically significant C: control group; SA: single arch group; FM: full mouth group; Lt: left; Rt: right

State	Group	N	Mean	SD	P-Value	Pairwise comparison	P-Value
Relax (R Muscle)	G1 (C)	10	2.45	1.85	0.15	C Vs SA	0.60
	G2 (SA)	10	1.32	0.70		C Vs FM	0.55
	G3 (FM)	10	3.70	4.05		SA Vs FM	0.15
Clench (R Muscle)	G1 (C)	10	5.95	3.29	0.86	C Vs SA	0.97
	G2 (SA)	10	5.27	0.95		C Vs FM	0.96
	G3 (FM)	10	6.51	7.93		SA Vs FM	0.85
Swallow (R Muscle)	G1 (C)	10	8.52	3.17	0.02*	C Vs SA	0.27
	G2 (SA)	10	6.02	3.34		C Vs FM	0.40
	G3 (FM)	10	10.35	2.98		SA Vs FM	0.01*
Rt. Side Chew (R Muscle)	G1 (C)	10	11.35	5.34	0.27	C Vs SA	0.65
	G2 (SA)	10	9.49	3.70		C Vs FM	0.24
	G3 (FM)	10	7.81	4.60		SA Vs FM	0.75
Lt. side Chew (R Muscle)	G1 (C)	10	10.25	5.69	0.35	C Vs SA	0.34
	G2 (SA)	10	7.60	2.55		C Vs FM	0.99
	G3 (FM)	10	9.99	3.87		SA Vs FM	0.47
Relax (L Muscle)	G1 (C)	10	4.95	3.35	0.02*	C Vs SA	0.02*
	G2 (SA)	10	2.45	1.09		C Vs FM	0.08
	G3 (FM)	10	2.75	1.15		SA Vs FM	0.82
Clench (L Muscle)	G1 (C)	10	7.99	4.28	0.39	C Vs SA	0.40
	G2 (SA)	10	5.63	2.10		C Vs FM	0.53
	G3 (FM)	10	6.05	4.71		SA Vs FM	0.99
Swallow (L Muscle)	G1 (C)	10	8.38	3.25	0.35	C Vs SA	0.75
	G2 (SA)	10	7.45	3.70		C Vs FM	0.73
	G3 (FM)	10	10.05	3.78		SA Vs FM	0.30
Rt. Side Chew (L Muscle)	G1 (C)	10	15.25	8.20	0.04*	C Vs SA	0.69
	G2 (SA)	10	12.95	4.15		C Vs FM	0.04*
	G3 (FM)	10	8.27	4.57		SA Vs FM	0.20
Lt. side Chew (L Muscle)	G1 (C)	10	10.35	4.60	0.88	C Vs SA	0.96
	G2 (SA)	10	10.99	2.85		C Vs FM	0.85
	G3 (FM)	10	11.65	7.15		SA Vs FM	0.99

However, there was no statistically significant difference amongst the three groups in the right digastric during the relaxed state, the right and left digastric muscle during MVC, the left digastric muscle during swallowing, the right digastric muscle during right side chewing, and the right and left digastric muscle activities during left side chewing.

## Discussion

Patients who have experienced tooth loss often seek rehabilitation in the hopes of having their replacement teeth perform as well as their original teeth did. Dentures supported by edentulous ridges cannot, however, offer this [10]. Today, individuals who are completely or partly toothless have a viable therapeutic option in implant-supported prostheses [11]. Restoring oral function with implant-supported prostheses is possible, and patients report more satisfaction with their chewing skills on both subjective and objective measures than they did with complete dentures [12]. Both healthy people and those with medical conditions are studied to report on the functional connections between the trigeminal, cervical, and upper limb systems [13]. Previous findings on the reaction of the SCM and digastric muscles during holding effort are confirmed by an enlarged EMG example of the muscles during MVC from a resting state, which reveals co-enactment of the front neck muscles during isometric withdrawal of the mandibular lift muscles [14].

Patients with varying degrees of prosthetic reconstructions and controls with intact dentitions were evaluated in the present study. All patients reported being happy with their prostheses and experiencing adequate masticatory efficiency. Subjects were divided into three groups: G1 (control; dentate), G2 (single arch implant-supported fixed prosthesis opposing natural dentition), and G3 (full mouth implant-supported fixed prosthesis). At least six months had passed after their prosthetic reconstructions had been completed, a period of time deemed sufficient for the establishment of good muscle activity and force production so that testing could begin [15]. Dental occlusion's neuromuscular balance may be affected by more than just the number of teeth and the configuration of dental contacts. No patients were found to have any dental connections between their arches, and everyone had a full set of occluding teeth (i.e., at least six pairs). The minimum number of teeth required for efficient eating is 10 units or 20 teeth in ideal alignment [16].

At a relaxed state, the temporalis muscle showed a significantly greater mean EMG action in people with full-mouth embedded upheld fixed prostheses compared to those with false teeth. The result of the present study, however, is analogous to the research done by Soni et al. [17] where the masseter muscle exhibited larger electromyographic activity compared to another muscle group, while in the present study, the digastric muscle showed a significantly greater mean EMG action in people with single-curve upheld fixed prostheses compared to those with false teeth. Mandibular resting posture is dependent on a delicate balancing act between the nerves, muscles, and bones in the face. Our findings show that sensory receptors in the neurological system regulate resting masticatory muscle tonic activity. The muscular tension exhibited in this posture was shown to shift if the stomatognathic system's homeostasis was disturbed, as shown by the current research [18].

At maximum voluntary clenching (MVC)

When comparing dentate individuals to those with single arch implant-supported fixed prostheses opposing natural teeth and complete mouth implant-supported fixed prostheses, the current study discovered that temporalis and masseter muscle activity was greater during MVC in the dentate group. Statistically speaking, no significant difference was seen between the groups. The highest amount of biting power may be achieved by a coordinated increase in the recruitment of motor units during clenching. With more depolarized motor units, the electromyograph will record a much larger potential. Dakhilalian et al. [19] in their study also reported decreased rate of the masseter and temporalis muscles while clenching after removing the attachments. Increase in muscular activity as well as bite force can be attributed to greater retention and stability of implant-supported overlay dentures (ISODs) in relation to conventional removable complete dentures (CRCD)s.

At swallowing

In the buccal phase of swallowing, the tongue is "spread and lifted" with the help of its extrinsic and intrinsic muscles such that the dorsum of the tongue makes contact with the palate, as further described by Rix. When the mylohyoids tighten, the tongue is pressed firmly against the roof of the mouth and the back of the teeth [20]. The current review found that the average EMG activity of the digastric muscle was greater in full-mouth embed upheld fixed prosthesis cases compared to those with a single-curve embed upheld fixed prosthesis and dentate individuals, with the difference between these two groups being particularly striking.

Unilateral chewing

Regarding unilateral chewing (right side), there was no discernible distinction between the three categories. Regarding unilateral chewing (left side), the results showed no statistically significant differences among the three groups. No significant changes were detected for the examined neck muscle; the SCM showed equal EMG activity and percentage of co-contraction during MVC regardless of the kind of occlusal support. Similar shifts in SCM activity were observed with similar shifts in occlusal surfaces; hence, these findings are consistent with earlier research [21,22]. Digastric muscular activity at maximal voluntary contraction was also similar in all three groups. Dakhilalian et al. in their study reported significant differences in masseter muscle activity while chewing (p = 0.03), indicating asymmetrical activity with CRCD. They also stated that the temporalis muscles showed asymmetrical activity with ISOD [19].

On the other hand, this research was not without its flaws. However, as stated by Furuyama et al., it is not always possible to conduct research with the best possible design [23]. The number of implants (four or six fixtures), the type of prosthesis (hybrid or porcelain-fused-to-metal (PFM)), and the number of occlusal contacts between opposing teeth were not considered in this study.

## Conclusions

The following inferences may be made in light of the data that was collected and analyzed statistically. With respect to the temporalis muscle, resting sEMG activity was shown to be significantly greater in the complete mouth implant-supported fixed prosthesis group than in the dentate and single arch implant-supported fixed groups. The sEMG analysis of the analyzed muscles during the static (clenching) task showed similar results in the three groups with a relative non-significant increase in the temporalis and masseter muscle activity. The sEMG activity during swallowing showed similar muscle activity amongst the three except the digastric muscle which had increased activity in the full mouth group. The sEMG activity during unilateral chewing showed similar patterns of muscular activity apart from masseter muscle activity on the working side which showed different activity patterns in the three groups. An increase in muscular activity, as well as bite force, can be attributed to greater retention and stability of the denture.
